# Unraveling genomic associations with feed efficiency and body weight traits in chickens through an integrative approach

**DOI:** 10.1186/s12863-019-0783-3

**Published:** 2019-11-06

**Authors:** Gabriel Costa Monteiro Moreira, Mirele Daiana Poleti, Fábio Pértille, Clarissa Boschiero, Aline Silva Mello Cesar, Thaís Fernanda Godoy, Mônica Corrêa Ledur, James M. Reecy, Dorian J. Garrick, Luiz Lehmann Coutinho

**Affiliations:** 1 Department of Animal Science, University of São Paulo, Piracicaba, SP 13418-900 Brazil; 2 University of São Paulo (USP) / College of Animal Science and Food Engineering (FZEA), Pirassununga, São Paulo, Brazil; 3 Embrapa Suínos e Aves, Concórdia, Santa Catarina Brazil; 4 Department of Animal Science, Iowa State University (ISU), Ames, Iowa, USA; 5 School of Agriculture, Massey University, Ruakura, Hamilton, New Zealand

**Keywords:** Genotypic data, GWAS, performance traits, Genomic heritability

## Abstract

**Background:**

Feed efficiency and growth rate have been targets for selection to improve chicken production. The incorporation of genomic tools may help to accelerate selection. We genotyped 529 individuals using a high-density SNP chip (600 K, Affymetrix®) to estimate genomic heritability of performance traits and to identify genomic regions and their positional candidate genes associated with performance traits in a Brazilian F_2_ Chicken Resource population. Regions exhibiting selection signatures and a SNP dataset from resequencing were integrated with the genomic regions identified using the chip to refine the list of positional candidate genes and identify potential causative mutations.

**Results:**

Feed intake (FI), feed conversion ratio (FC), feed efficiency (FE) and weight gain (WG) exhibited low genomic heritability values (i.e. from 0.0002 to 0.13), while body weight at hatch (BW1), 35 days-of-age (BW35), and 41 days-of-age (BW41) exhibited high genomic heritability values (i.e. from 0.60 to 0.73) in this F_2_ population. Twenty unique 1-Mb genomic windows were associated with BW1, BW35 or BW41, located on GGA1–4, 6–7, 10, 14, 24, 27 and 28. Thirty-eight positional candidate genes were identified within these windows, and three of them overlapped with selection signature regions. Thirteen predicted deleterious and three high impact sequence SNPs in these QTL regions were annotated in 11 positional candidate genes related to osteogenesis, skeletal muscle development, growth, energy metabolism and lipid metabolism, which may be associated with body weight in chickens.

**Conclusions:**

The use of a high-density SNP array to identify QTL which were integrated with whole genome sequence signatures of selection allowed the identification of candidate genes and candidate causal variants. One novel QTL was detected providing additional information to understand the genetic architecture of body weight traits. We identified QTL for body weight traits, which were also associated with fatness in the same population. Our findings form a basis for further functional studies to elucidate the role of specific genes in regulating body weight and fat deposition in chickens, generating useful information for poultry breeding programs.

## Background

Poultry breeding programs were developed to increase growth rate, body condition (performance traits) and improve carcass traits in broiler chickens [1, 2]. Nevertheless, selection to increase productivity, reduce production costs and minimize environmental impact remains a challenge for chicken breeders. Feed efficiency and body weight are the two main performance traits with economic importance [3] that may drive increased chicken productivity. The incorporation of genomic tools in breeding programs can increase genetic progress by improving selection accuracy [4, 5]. Additionally, the chicken is considered an important model for animal genomic studies [6]. Thus, the search for genomic regions and positional candidate genes can help to elucidate the molecular mechanisms involved in the regulation of performance traits not only in chickens, but also in other species.

The Chicken QTL database (release 35) [7] hosts 75 quantitative trait loci (QTL) for feeding traits (including feed conversion ratio, feed efficiency, feed intake, and residual feed intake). In contrast, 1637 QTL have been reported for growth traits (including average daily gain and body weight at different days of age). However, many of these QTL have only been coarsely mapped (i.e., they have very broad confidence intervals for location). QTL mapping studies were previously conducted on this Brazilian experimental population (Embrapa F_2_ Chicken Resource Population) for feeding, growth and carcass traits [8, 9]. However, those studies utilized up to 127 microsatellite markers, which resulted in the identification of QTL that span large regions of the genome. The average length of interval of the QTL reported by Nones et al. [9] and Ambo et al. [8] were 5.46 Mb and 11.9 Mb, respectively.

Recent studies have identified QTL, candidate genes and mutations associated with performance traits in chickens [3, 10, 11]. Yi et al. [11] investigated SNPs associated with performance traits in a Chinese local chicken population and identified two SNPs in the *CCKAR* gene associated with daily feed intake and daily gain. Mebratie et al. [3] identified 11 QTL and 21 SNPs associated with body weight traits, and 5 QTL and 5 SNPs associated with feed efficiency traits in a commercial broiler chicken population. Despite these recent efforts, the re-visitation of genome-wide association study (GWAS) for performance traits, using a higher density of markers, may enable the identification of genomic regions with smaller intervals [12], thereby facilitating the fine-mapping of novel and known QTL. This can aid in the identification of positional candidate genes and, eventually, the identification of potentially causative mutations [12, 13].

Recently, GWAS for performance traits in Embrapa F_2_ Chicken Resource Population was performed using 134,528 SNPs generated from a genotyping by sequencing (GBS) approach that used the restriction endonuclease *PstI* [10]. Although that study identified 21 SNPs that were significantly associated with the performance traits, the use of a high-density SNP array to genotype the same population may provide more uniform coverage of regions across the whole chicken genome.

The aims of this study were to estimate the genomic heritability for performance traits, and to identify genomic regions and positional candidate genes associated with these traits in a Brazilian F_2_ Chicken Resource population that was derived from a reciprocal cross between a broiler and a layer line. In addition, selection signature regions and a SNP dataset derived from re-sequencing of grandparental individuals were integrated to refine the list of candidate genes and the search for potential causative mutations.

## Results

### Descriptive statistics

The number of animals, means and standard errors, variance components, and estimated genomic heritabilities are given in Table 1 for: feed intake between 35 and 41 days of age (FI), feed conversion ratio between 35 and 41 days of age (FC), feed efficiency between 35 and 41 days of age (FE), weight gain between 35 and 41 days of age (WG), body weight at hatch (BW1), body weight at 35 days of age (BW35) and body weight at 41 days of age (BW41). Genomic heritability values ranged from 0.0002 for FI to 0.73 for BW41.

**Table 1:** Descriptive statistics, variance components and genomic heritability

Trait	N	mean ± SD	Genetic Variance (SE)	Residual Variance (SE)	Total Variance (SE)	Genomic Heritability (SE)
FI	479	597.89 ± 132.88	1.4024 (1.8247)	8030.8300 (526.2320)	8032.2400 (526.2090)	0.0002 (0.0002)
FC	472	2.84 ± 0.74	0.0333 (0.0067)	0.4429 (0.0311)	0.4760 (0.0311)	0.07 (0.0143)
FE	471	0.37 ± 0.07	0.0006 (0.00009)	0.004 (0.0003)	0.0045 (0.0003)	0.13 (0.0220)
WG	459	220.00 ± 67.25	2.6996 (0.8366)	217.5120 (14.7378)	220.2120 (14.7127)	0.01 (0.0039)
BW1	478	44.57 ± 4.49	5.1527 (0.3810)	3.3557 (0.3113)	8.5084 (0.4336)	0.60 (0.0313)
BW35	480	790.92 ± 140.53	8511.6100 (544.2520)	3356.1100 (414.5550)	11,867.7000 (521.7490)	0.72 (0.0328)
BW41	480	1009.43 ± 190.74	15,430.8000 (975.2460)	5835.5000 (739.7160)	21,266.3000 (917.5350)	0.73 (0.0330)

*FI* Feed intake between 35 and 41 days of age, *FC* Feed conversion ratio between 35 and 41 days of age, *FE* Feed efficiency between 35 and 41 days of age, *WG* Weight gain between 35 and 41 days of age, *BW1* Body weight at hatch, *BW35* Body weight at 35 days of age, *BW41* Body weight at 41 days of age. *SD* is the standard deviation and *SE* is the standard error

### Genotyping and genome-wide association studies

As described by Moreira et al. [14], from the 529 genotyped chickens, 12 were removed from the analysis after applying animal DishQC criteria and a sample call rate filter ≥90%. The 28 grandparental chickens and 12 F_1_ birds did not have phenotypic data and were not considered for GWAS. A total of 489 F_2_ chickens from seven different families were used in the association analysis.

From the 580,961 SNPs on the SNP chip array, 399,693 segregating SNPs were kept for further analyses. All these SNPs had a call rate ≥ 98%. Among these, 4304 were removed due to minor allele frequency (MAF) criteria (MAF ≤ 0.02), and 23,603 SNPs that were located on the sex chromosomes and linkage groups were also removed, such that 371,558 markers remained for GWAS. An average density of 541 SNPs/Mb per chromosome was observed, with the lowest density on GGA2 (297 SNPs/Mb) and the highest density on GGA21 (816 SNPs/Mb). Missing genotypes were replaced with the average covariate value of that locus as reported by Cesar et al. [15].

The characterization of the 943 1-Mb non-overlapped windows and their respective percentage of the genetic variance explained are available in Additional file 1. The genomic windows associated with performance traits are described in Table 2. Twenty unique 1-Mb windows on GGA1–4, 6, 7, 10, 14, 24, 27 and 28 were associated with the body weight traits. The posterior probability of association (PPA) for these regions ranged from 0.40 to 0.96 and the genetic variance explained by each SNP window ranged from 0.53 to 4.74%. We did not identify any genomic windows associated with FI, FC, FE, or WG.

**Table 2:** Characterization of 1-Mb genomic windows that explained more than 0.53% of the genomic variance for body weight traits

Trait	GGA_Mb	Genomic window (first and last SNP)	#SNPs	Percentage of genetic variance explained	PPA^1^
BW1	1_181	rs14928423 - rs314828711	388	1.45	0.65
	6_2	rs317072624 - rs14561583	461	0.67	0.50
BW35	1_54	rs15271198 - rs315312994	257	0.85	0.48
	1_55	rs315667199 - rs314256540	223	0.66	0.44
	1_56	rs317748170 - rs15279198	411	0.94	0.63
	1_129	rs312987852 - rs312615910	385	0.81	0.58
	1_168	rs318211853 - rs15497155	318	3.08	0.82
	2_78	rs318038016 - rs314335165	282	0.92	0.53
	3_28	rs313517177 - rs313321588	342	0.53	0.50
	3_30	rs317825887 - rs13722119	365	1.42	0.62
	4_69	rs14487157 - rs314272956	367	0.75	0.50
	4_74	rs316224092 - rs317555947	281	0.75	0.40
	4_76	rs15618974 - rs314892344	308	3.26	0.64
	7_34	rs316467562 - rs312928601	411	0.57	0.63
	7_36	rs316261866 - rs315360554	257	0.60	0.49
	14_9	rs315659517 - rs317168690	703	0.69	0.69
	24_1	rs316118891 - rs14293772	814	0.73	0.82
	27_3	rs14302748 - rs312772391	820	1.93	0.94
	28_0	rs313774457 - rs312701176	829	2.10	0.92
BW41	1_54	rs15271198 - rs315312994	257	0.69	0.50
	1_56	rs317748170 - rs15279198	411	0.89	0.58
	1_168	rs318211853 - rs15497155	318	2.33	0.75
	2_78	rs318038016 - rs314335165	282	0.70	0.51
	3_30	rs317825887 - rs13722119	365	1.26	0.66
	4_74	rs316224092 - rs317555947	281	1.20	0.49
	4_76	rs15618974 - rs314892344	308	4.74	0.74
	10_16	rs14011271 - rs313957691	623	0.72	0.74
	27_3	rs14302748 - rs312772391	820	1.75	0.92
	28_0	rs313774457 - rs312701176	829	3.03	0.96

^1^Posterior probability of association (PPA) as reported by Onteru et al. [16]

**Fig. 1:**
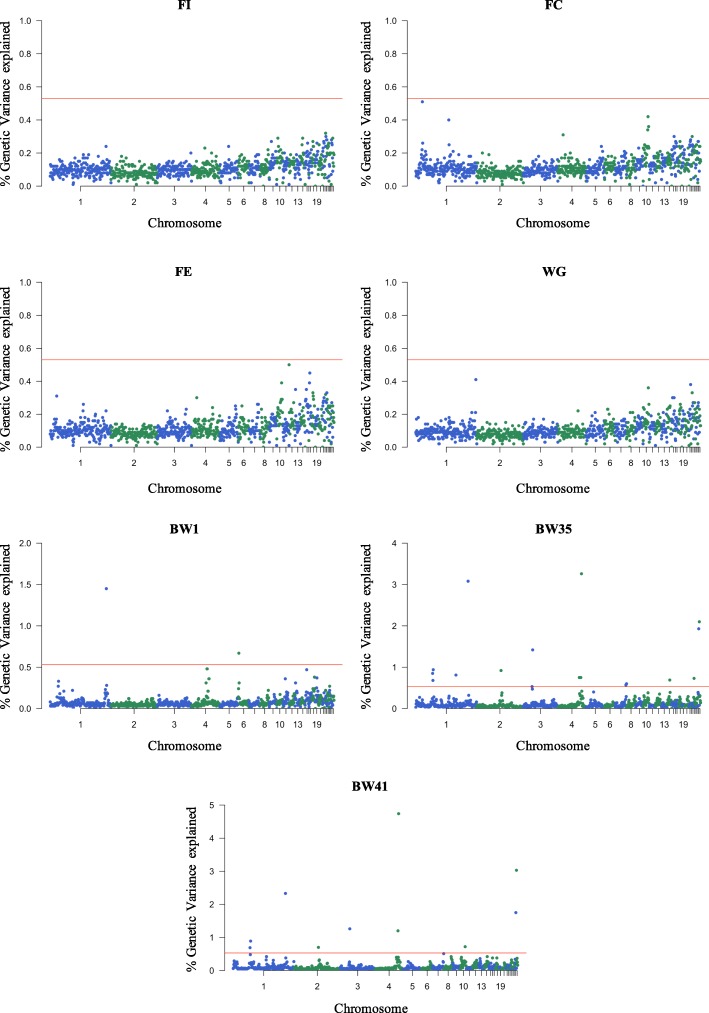
Manhattan plots of the posterior means of the percentage of genetic variance explained by each 1 Mb SNP window across the 28 autosomal chromosomes for all the performance traits analyzed. The title of each graph indicates the corresponding phenotype: feed intake (FI), feed conversion (FC), feed efficiency (FE), weight gain (WG), body weight at hatch (BW1); body weight at 35 days of age (BW35); body weight at 41 days of age (BW41). The X-axis represents the ordered chromosomes, and Y-axis shows the proportion of genetic variance explained by each window from Bayes B analysis. Red lines indicate the threshold to deem significant SNP windows (0.53%)

The associated SNP windows cumulatively explained 2.12, 20.59, and 17.31% of the genetic variance for BW1, BW35 and BW41, respectively. Manhattan plots with the percentage of genetic variance explained by all 943 non-overlapped SNP windows for each trait analyzed herein are shown in Fig. 1.

From the 20 unique genomic windows (Table 2) associated with body weight traits, two genomic windows were associated exclusively with BW1, eight with BW35, one with BW41 and nine were associated only with BW35 and BW41 (Table 2). Within these latter nine genomic windows associated with BW35 and BW41, SNPs with the highest model frequency were investigated to identify whether the same or different SNPs accounted for the genetic variance explained by the window for BW35 and BW41. The characterization of the SNPs with the highest model frequency are shown in Table 3.

**Table 3:** Characterization of SNPs with the highest model frequency within the nine genomic windows associated with BW35 and BW41

Genomic windows associated	BW35		BW41	
	SNP ID^1^	Model Frequency	SNP ID^1^	Model Frequency
1_54	rs315625251	0.0154	rs315625251	0.0142
1_56	rs13871363	0.0174	rs315430937	0.0200
1_168	rs14916269	0.0708	rs316630786	0.1002
2_78	rs314546937	0.0119	rs314546937	0.0071
3_30	rs313673308	0.0355	rs312452371	0.0432
4_74	rs315474450	0.0157	rs315474450	0.0262
4_76	rs315283155	0.0593	rs314495350	0.0811
27_3	rs16719146	0.0329	rs80711851	0.0234
28_0	rs14305335	0.1893	rs14305335	0.3252

^1^SNP within the window with the highest model frequency

### Overlapping with known QTL

From the 20 unique genomic windows (Table 2) associated with body weight traits, 19 overlapped with previously published QTL for body weight traits curated in the Chicken QTL database (release 35). Among those, nine overlapped with QTL associated with exactly the same traits that had been mapped in this same population in previous studies using microsatellite markers [8, 9]. The overlaps are available in the Additional file 2. The genomic window located on GGA24 is a novel QTL, since it did not overlap with any previously published QTL region.

From the 20 unique genomic windows associated with body weight traits (Table 2), five overlapped with QTL previously mapped for fatness traits using the same SNP dataset and the same population (Embrapa F_2_ Chicken Resource Population) [14] (Table 4).

**Table 4:** Genomic windows that overlapped with QTL previously mapped for fatness traits using the same SNP dataset and the same population (Embrapa F_2_ Chicken Resource Population)

GGA_Mb	Genomic window	Genome interval	Associated trait herein	Fatness associated trait [14]
	(first - last SNP)	(start – end position)^1^		
1_54	rs318211853 - rs15497155	54,001,671 – 54,998,619	BW35, BW41	ABF
1_168	rs15271198 - rs315312994	168,005,668 – 168,997,872	BW35, BW41	CFC
7_36	rs14302748 - rs312772391	36,000,235 – 36,898,384	BW35	CFC, CFCDM
27_3	rs313774457 - rs312701176	3,000,222 – 3,996,811	BW35, BW41	ABF
28_0	rs316261866 - rs315360554	23,942 – 999,295	BW35, BW41	ABFP

*ABF* Abdominal fat weight in grams, *ABFP* Abdominal fat percentage, *CFC* Carcass fat content in grams, *CFCDM* Carcass fat content on dry matter basis

^1^Map position based on Gallus_gallus-5.0, NCBI assembly

### Positional candidate genes

Our enrichment analysis did not identified MeSH terms associated with any of the categories investigated (i.e, Chemical and Drugs, Diseases, Anatomy, Phenomena and Processes) and also, no enriched clusters of genes were detected. Thus, positional candidate genes within each genomic window were evaluated. From the 386 genes annotated within the associated genomic windows (Additional file 3), 38 were selected as possible candidates for body weight regulation in chickens, based on their respective biological GO terms and literature information (Table 5).

**Table 5:** Genomic windows associated with body weight traits and their positional candidate genes, and overlap with selection signature regions

GGA (Pos Mb)	Trait	PCG^1^	Ensembl gene ID^2^
1 (54)	BW35, BW41	CHST11**	ENSGALG00000030607
		*TXNRD1*	ENSGALG00000035345
		*SLC41A2*	ENSGALG00000012697
1 (55)	BW35	*PMCH*	ENSGALG00000012757
1 (56)	BW35, BW41	*HIPK2*	ENSGALG00000012792
		*AKR1D1*	ENSGALG00000012834
		*SLC37A3*	ENSGALG00000012849
1 (129)	BW35	*SLC25A6*	ENSGALG00000016691
1 (168)	BW35, BW41	*RB1*	ENSGALG00000016997
		*HTR2A*	ENSGALG00000016992
3 (30)	BW35, BW41	*SLC29A1*	ENSGALG00000010182
		*HSP90AB1*	ENSGALG00000010175
		*VEGFA*	ENSGALG00000010290
4 (69)	BW35	*RBM47*	ENSGALG00000014267
		*CHRNA9*	ENSGALG00000014268
		*KLB*	ENSGALG00000041663
4 (74)	BW35, BW41	*PPARGC1A*	ENSGALG00000042851
4 (76)	BW35, BW41	*LCORL*	ENSGALG00000014421
6 (2)	BW1	*BMPR1A*	ENSGALG00000002003
7 (34)	BW35	ACVR2A**	ENSGALG00000012444
7 (36)	BW35	*ACVR1C*	ENSGALG00000041257
		*ACVR1*	ENSGALG00000037301
		NR4A2**	ENSGALG00000012538
10 (16)	BW41	*IGF-1R*	ENSGALG00000040651
14 (9)	BW35	*EMP2*	ENSGALG00000027058
		*GRIN2A*	ENSGALG00000007278
24 (1)	BW35	*KCNJ5*	ENSGALG00000001181
		*KCNJ1*	ENSGALG00000001167
27 (3)	BW35, BW41	*HOXB7*	ENSGALG00000032740
		*HOXB2*	ENSGALG00000025774
		*HOXB13*	ENSGALG00000033154
		*HOXB9*	ENSGALG00000001276
		*HOXB4*	ENSGALG00000000284
		*PHB*	ENSGALG00000038604
		*SLC35B1*	ENSGALG00000035057
28 (0)	BW35, BW41	*ANGPTL4*	ENSGALG00000000619
		*SLC1A6*	ENSGALG00000000558
		*LONP1*	ENSGALG00000040492

**Positional candidate genes which overlapped with selection signature regions [17]

^1^Positional candidate genes

^2^Ensembl gene ID based on Galgal5 (Ensembl release 92)

In addition, positional candidate genes that were located within regions under selection previously identified in the grandparental chicken lines used to generate the F_2_ population [17] were further investigated. The list of genomic windows that harbored positional candidate genes and their overlap with signature selection regions are shown in Table 5.

### Search for SNPs in positional candidate genes

Sixteen functional SNPs (13 predicted as deleterious and three as high impact SNPs) were annotated in 11 positional candidate genes (Table 6), integrating the sequence SNPs detected by the re-sequencing of grandparental ancestral chickens used to produce the F_2_ population.

**Table 6:** Characterization of predicted deleterious and high impact SNPs annotated in 11 positional candidate genes

Gene	GGA	SNP ID	Position^1^	Annotation	SIFT score	AA substitution
*AKR1D1*	1	*rs316370743*	56,636,977	Deleterious	0.01	Met/Ile
*HSP90AB1*	3	*rs737959833*	30,358,254	Deleterious	0.03	Thr/Ala
		*rs737623405*	30,357,799	High impact (Stop lost)	–	*/Arg
*KLB*	4	*rs740538348*	69,722,817	Deleterious	0.02	Arg/Trp
*RBM47*	4	*rs313177163*	69,358,984	Deleterious	0.00	Arg/Cys
*PPARGC1A*	4	*rs739990319*	74,565,856	Deleterious	0.00	Arg/Gly
		*rs16435584*	74,566,888	Deleterious	0.01	Asp/Asn
		*rs731752899*	74,590,596	Deleterious	0.01	Asn/Asp
*NR4A2*	7	*g.36224286C > T (Novel)*	36,224,286	Deleterious	0.00	Val/Met
		*g.36225242G > T (Novel)*	36,225,242	Deleterious	0.00	Arg/Ser
		*g.36225278C > T (Novel)*	36,225,278	Deleterious	0.01	Val/Met
*ACVR1*	7	*rs312541186*	36,479,417	Deleterious	0.01	Trp/Arg
*GRIN2A*	14	*rs316241099*	9,451,676	High impact (Splice acceptor)	–	–
*KCNJ5*	24	*rs312300440*	1,075,890	Deleterious	0.02	Leu/Pro
*SLC35B1*	27	*g.3338981C > T (Novel)*	3,338,981	High impact (Stop gained)	–	Gln/*
*ANGPTL4*	28	*g.846035G > A (Novel)*	846,035	Deleterious	0.03	Ser/Phe

^1^Position based on assembly Gallus_gallus-5.0

## Discussion

### Genomic heritability

The genomic heritability estimates ranged from 0.0002 to 0.13 (Table 1) for FI, WG, FC, and FE. These are complex traits, which are also subject to several environmental factors and, as a result, commonly exhibit low heritability estimates [18]. Moreover, these traits were measured only between 35 and 41 days in an F_2_ population that exhibits high phenotypic and genotypic variability, and were adjusted using BW35 as a covariate. The short interval between the two measurements could explain the low genomic heritability estimates observed [8]. Nevertheless, FI, FC, FE, and WG are extremely important traits that influence the costs of production and, therefore, should be considered in breeding programs.

Different heritability estimates were found in the literature based on the use of pedigree records to define the genetic variance-covariance among animals, as reported by Gaya et al. [19], who obtained 0.20 for FI and 0.16 for FC (from 5 to 7 weeks of age) using one single sire broiler population. FI, FC (both measured between 35 and 42 days of age) and WG were reported by Aggrey et al. [20] to exhibit values of 0.46, 0.41 and 0.48, respectively, using one random bred population. For body weight traits, Venturini et al. [21] obtained 0.41 for heritability of body weight at 42 days-of-age (BW42) and Grupioni et at [22]. obtained 0.50 for BW42, both using broiler populations.

Using the genomic relationship matrix, Mebratie et al. [23] found heritability estimates of 0.090 for FI, 0.051 for FC and 0.027 for WG (measured in an interval of 7 days of age), in a male broiler population. Abdollahi-Arpanahi et al. [24] obtained heritability of 0.30 for BW35, using a commercial broiler population. It is important to highlight that these studies have used broiler populations that have been under artificial selection. Artificial selection may affect genetic variance and, consequently, genomic heritability [25].

In contrast, this study was performed using an F_2_ chicken population derived from a cross between divergent lines. Thus, differences in genomic heritability estimates should be expected due to the genetic variability between the lines which introduces segregation variance into the F_2_. Another study using the same F_2_ population and a lower density of markers (~ 135 K, Cornell GBS approach), Pértille et al. [10] reported genomic heritability for FC (0.01 ± 0.006), FE (0.11 ± 0.005), FI (0.17 ± 0.094), BW1 (0.45 ± 0.073), BW35 (0.85 ± 0.073) and BW41 (0.75 ± 0.087). Differences in the genomic heritability estimates, even in the same population, are expected due to sampling errors, but can be magnified due to the presence of large effect QTL in the dataset utilized.

### Genomic windows identified

The genetic variance explained by each SNP window associated with body weight traits ranged from 0.53 to 4.74%, with windows located on GGA1–4, 6, 7, 10, 14, 24, 27 and 28. Ninety-five percent (19 out of 20) of the genomic windows identified overlapped with at least two known QTL for body weight traits (Additional file 2). Additionally, nine genomic windows on GGA3, 4, 7, 10 and 27 overlapped with known QTL mapped for the same trait in the same population [8] (Additional file 2). Although 95% of the detected genomic windows were already known to be associated with body weight traits as reported in the Chicken QTL database [7], some of them were associated, for the first time, with body weight at the ages analyzed herein. These results provide valuable information to understand the genetic control of body weight, a major factor involved in chicken growth and development.

We also compared genomic window sizes with the span of known QTL previously mapped for the same trait in the same population [8] (Additional file 2). These known QTL were previously mapped using QTL interval mapping (linkage analysis) with up to 127 microsatellite markers, covering 21 linkage groups [8]. Most of our genomic windows had fixed 1 Mb width, with the exception of those windows associated with the same trait that were located in an immediately flanking position, which were merged and the wider combined window was considered as a unique window. Considering this approach, we were able to refine up to 99% of the size of the known QTL (QTL: 7180, 100.4 Mb of size; Additional file 2). Thus, the use of a high density of markers to perform GWAS provided us with much better resolution for QTL detection, facilitating the search for positional candidate genes.

From the 20 unique genomic windows associated with body weight traits (Table 2), five overlapped with QTL previously mapped for fatness traits using the same SNP dataset and the same population (Embrapa F_2_ Chicken Resource Population) [14] (Table 4). Thus, we suggest that these QTL may exhibit pleiotropic effects, affecting different tissues (such as adipose tissue) and metabolic processes associated with body weight regulation in chickens.

Two genomic windows were associated exclusively with BW1, nine with BW35, one with BW41, while eight were associated with both BW35 and BW41 (Table 2). From these nine genomic windows, four exhibited the same SNP with the highest model frequency for BW35 and BW41. The different percentages explained by the same QTL at different ages may be related to changes in metabolic processes regulating body weight during chicken growth and development. Four genomic windows associated with both BW35 and BW41 exhibited different SNPs with the highest model frequency (Table 3). Among those QTL, the windows on GGA1 at 168 Mb, GGA7 at 36 Mb, and GGA27 at 3 Mb overlapped with QTL previously mapped for fatness traits using the same SNP dataset and the same population (Table 4). Thus, these findings corroborate that different genes, tissues (such as adipose tissue) and metabolic processes can be involved in the regulation of body weight in chickens. Further studies to quantify positional candidate gene expression at different ages could be helpful.

The genomic window on GGA24 did not overlap with published QTL for body weight traits and was considered as a novel QTL. This QTL might be population specific, thus, further studies in other populations might be helpful to validate the role of this region in body weight regulation.

As mentioned earlier, we did not identify any genomic windows associated with FI, FC, FE or WG. This could be due to the lower genetic variance detected for these traits compared to those reported for the other traits (BW1, BW35 and BW41) (Table 1).

### Positional candidate genes for body weight in chickens

Within the associated genomic windows, 38 genes were selected as candidates for body weight regulation in chickens, based on their respective biological GO terms and literature information (Table 5). Three of those have already been associated with carcass and body weight traits in livestock - *LCORL* [26, 27], *PPARGC1A* [28, 29] and *CHRNA9* [30]. Fifteen genes were previously associated with growth and development processes, such as cell growth and proliferation (*HOXB2, 4, 7, 9 and 13, HIPK2* [31]*, KLB* [32–34] and *PHB* [35, 36]); embryo development and early growth (*TXNRD1* [37], *IGF-1R* [38], *RBM47* [39] and *VEGFA* [40, 41]) or muscle hypertrophy and development (*LONP1* [42]*, GRIN2A* [43] and *BMPR1A* [44]). It is important to highlight that *HOXB2, 4, 7, 9 and 13* gene*s* belong to the homeobox family, known to be associated with development [45] and stem cell growth and differentiation [33]. In addition, the interaction between VEGF members (such as *VEGFA*) and their receptors may promote cell differentiation in various tissues, such as skeletal muscle in mammals.

One positional candidate gene was associated with heat stress: *HSP90AB1.* The *HSP90AB1* gene encodes a heat shock protein (HPS), which was associated with the response to heat stress in cattle [46, 47] and in general livestock adaptation [48]. Interestingly, heat stress has a negative impact on performance parameters in chickens [49, 50], corroborating that the *HSP90AB1* gene can affect performance traits and, consequently, body weight in chickens.

Three actin A receptor types were identified as positional candidate genes: *ACVR1*, *ACVR1C* and *ACVR2A*. The *ACVR1* gene is associated with ossification and its expression can inhibit osteogenesis [51, 52], potentially affecting body weight. The *ACVR1C* gene was associated with adiposity and body weight in mice [53]. The *ACVR2A* was associated with breast and carcass weight in chickens [54]. Additionally, the *ACVR2A* gene overlapped with one signature selection region previously identified in the founders of the Embrapa F_2_ Chicken Resource Population [17], which indicates that this gene was under positive selection affecting breast and carcass weights and, consequently, body weight in either the broiler or layer line.

Breeders have long been selecting chickens for rapid growth, body weight gain, feed efficiency and breast muscle weight [55–57]. Selection has resulted in chickens with higher growth rate and breast meat yield, as well as higher fat deposition [56]. Accordingly, in identifying candidates, we did not limit our consideration to only those genes with roles in cell differentiation and proliferation, skeletal muscle growth and development, but also considered genes with roles in adipose tissue development, or energy and lipid metabolism.

Fifteen positional candidate genes were associated with adipose tissue development, energy and lipid metabolism: *SLC41A2, SLC37A3, SLC25A6, SLC29A1, SLC1A6, SLC35B1, AKR1D1, ANGPTL4* [58]*, RB1* [59, 60]*, CHST11* [61]*, PMCH* [62]*, NR4A2* [63]*, HTR2A* [64]*, KCNJ5* and *KCNJ1.* It is important to highlight that *SLC41A2, SLC37A3, SLC25A6, SLC29A1, SLC1A6* and *SLC35B1* belong to the solute carrier family already known to be associated with energy metabolism [36, 65–67] and obesity in humans [68, 69]. The *AKR1D1* gene is involved with bile acid and steroid hormone homeostasis [70] and, interestingly, effects of dietary supplemental bile acids have already been associated with the activity of intestinal and lipoprotein lipases affecting growth performance in chickens [71]. The *KCNJ5* and *KCNJ1* genes belong to the potassium channel family, that may affect food intake, energy expenditure and glucose homeostasis [48] and, consequently, body weight.

From all the positional candidate genes identified, six were located within two QTL previously mapped for fatness traits [14] (Table 4), and were selected as candidates by Moreira et al. [14] for fat deposition regulation in the same population studied herein: *CHST11, RB1, HTR2A, NR4A2, ANGPTL4* and *SLC1A6*, suggesting that these genes may have pleiotropic effects. Those regions associated with body weight and fatness traits may help to explain why selection for weight gain is associated with increased fat deposition. Moreover, *CHST11* and *NR4A2* genes overlapped with a signature selection region previously identified in the founders of the Embrapa F_2_ Chicken Resource Population [17]. These result indicates that these genes are under positive selection and could help to explain the difference in fat deposition observed in the CC and TT lines used in the study. These findings provide helpful information for poultry breeding programs that aim to select birds with both high body weight and reduced fat deposition.

### Predicted deleterious and high impact SNPs

Thirteen predicted deleterious and three high impact SNPs were identified in 11 positional candidate genes from our gene list (Table 6). As previously mentioned, these genes were related to energy homeostasis, lipid metabolism and, consequently, body weight regulation. Moreover, the *NR4A2* gene overlapped with a selection signature region, indicating that this gene is under positive selection, may affecting lipid metabolism in one of the founder lines and, consequently, body weight. Thus, predicted deleterious and high impact SNPs in these genes could be causative mutations.

In summary, we identified 20 unique 1-Mb genomic windows associated with body weight traits (19 already known and one novel QTL) and within them, we detected 38 positional candidate genes. Through our integrative approach, we refined our list of candidate genes investigating the overlap between sequence SNPs and signatures of selection detected in the founders of the population. Curiously, three positional candidate genes overlapped with regions exhibiting selection signatures. In addition, thirteen predicted deleterious and three high impact SNPs were annotated in 11 positional candidate genes related to osteogenesis, skeletal muscle development, growth, energy metabolism and lipid metabolism, which may be associated with body weight in chickens. Further functional studies need to be performed to validate the role of these mutations in body weight regulation, thus providing important information for poultry breeding.

## Conclusions

The use of a high-density SNP array to identify QTL in an F_2_ population and the integration of regions exhibiting signatures of selection in their pure line ancestors along with sequence SNPs detected in pure line grandparents allowed the identification of candidate genes and candidate causal variants within those genes. Annotation of candidate genes indicates the importance of osteogenesis, cell growth and differenciation, skeletal muscle development, energy metabolism and lipid metabolism in the control of growth and, consequently, body weight in chicken. Our findings form a basis for further functional studies that can elucidate the role of specific genes in body weight regulation in chickens, generating useful information for poultry breeding programs.

## Methods

All experimental protocols related to animal experimentation in this study were performed in agreement with resolution number 010/2012 approved by the Embrapa Swine and Poultry National Research Center Ethics Committee on Animal Utilization to ensure compliance with international guidelines for animal welfare.

### Animals, population and phenotypes measured

We used the same population described in Moreira et al. [14]. In addition, the population used in this study is the same previously utilized to map QTL for performance, carcass, chemical components and organs traits using microsatellite markers [8, 9, 72–75]. Sires that exhibited favorable QTL effects previously mapped for those traits, had their progenies selected for high-density genotyping and genome-wide association.

In summary, 529 chickens from an Embrapa F_2_ Chicken Resource Population (developed by the Embrapa Swine and Poultry National Research Center) were genotyped (28 grandparental chickens from layer and broiler lines, 5 chickens from F_1_ and 496 chickens from the F_2_-TCTC generations) [14] with a high-density SNP array (600 K) [76].

Breifly, the layer line (CC) was selected for eight generations for improved egg production, egg weight, feed conversion, viability, sexual maturity, fertility, hatchability, egg quality, and low body weight, prior to the F_2_ population being created [14]. The broiler line (TT) had been selected for six generations, mainly for improved body weight, feed conversion, carcass and breast yield, viability, fertility, hatchability, lower abdominal fat weight, and reduced metabolic syndromes [14]. More details about the Embrapa F_2_ Chicken Resource Population are described by Nones et al. [9] and Rosário et al. [77].

Chickens from the F_2_ population were reared with free access to water and a corn and soybean meal-based diet up to 42 days of age [8]. As described by Ambo et al. [8] and Pértille et al. [10], between 35 and 41 days-of-age, chickens were transferred to individual cages for feed intake (FI) measurement and to compute feed conversion (FC), feed efficiency (FE) and weight gain (WG). Body weight was measured in grams (g) at hatch, 35 and 41 days of age (BW1, BW35, and BW41). The BW41 was collected at the end of the feed conversion test. WG was calculated as the difference between BW41 and BW35. FC was calculated by dividing FI by WG. FE was calculated by dividing WG by FI. Chickens were euthanized by cervical dislocation.

### DNA extraction, genotyping and quality control

The DNA extraction, genotyping and quality control have been described in Moreira et al. [14]. Briefly, genomic DNA was extracted from blood with DNAzol® following manufacturer recommendations (Life Technologies Invitrogen). After extraction, DNA integrity was evaluated in agarose gel (1%), quantified in NanoDrop® 2000 spectrophotometer (Thermo Fisher Scientific), then diluted to a final concentration of 20 ng.μL^− 1^. Diluted genomic DNA was prepared for genotyping following an Affymetrix protocol, and then genotyped with a 600 K Affymetrix Axiom Chicken Genotyping Array (Affymetrix, Inc. Santa Clara, CA, USA). That SNP chip contains segregating SNP for different chicken lines as described by Kranis et al. [76].

Quality control analysis and genotype calling were performed using Affymetrix Power Tools v1.17.0 (APT). Samples that exhibited DishQC ≥0.82 and call rates ≥90% were kept for further analyses. Filtering was performed with the SNPolisher package using R software (http://www.r-project.org/), and SNPs with call rate ≥ 98% and minor allele frequency (MAF) ≥ 2% were kept for further analyses. SNPs monomorphic, located on the sex chromosomes or linkage groups without genomic annotation were removed.

### Descriptive statistics, heritability and genome-wide association studies

The SNPs retained after filtering for quality were investigated in GWAS using genomic prediction methodology with a Bayesian approach in GenSel software [78]. In this approach, the genotypes are simultaneously fitted in the model which has been shown to account for any structure in the population [78, 79]. Previous studies have used this approach to perform GWAS and discover QTL and positional candidate genes in chickens based on high density markers [14, 80–83].

In a first step, BayesC was used to estimate the genetic and residual variances (with π =0). Those values were then used to run a BayesB model, to estimate genomic heritability and perform GWAS, as had been adopted by Cesar et al. [15] and Moreira et al. [14, 83]. The mathematical model presented below was used in the association analyses:

$$ \boldsymbol{y}=\boldsymbol{Xb}+\sum \limits_{j=1}^k{\boldsymbol{a}}_j{\beta}_j{\delta}_j+\boldsymbol{e}, $$

In this model, ***y*** represents a vector of phenotypic values, ***X*** an incidence matrix for fixed effects, ***b*** the vector of fixed effects, *k* the number of SNP, **a**_*j*_ the column vector representing SNP locus _j_ as a covariate coded with the number of B alleles, *β*_*j*_ the random substitution effect for locus *j* assumed normally distributed *N* (0, *σ*^2^_*β*_) when *δ*_*j*_ = 1, with *δ*_*j*_ being a random indicator variable 0/1, indicating the absence (with probability π) or presence (with probability 1-π) of locus *j* in the model, and *e* the vector of the residual effects assumed normally distributed *N* (0, *σ*^2^_*e*_). Sex and hatch were included as fixed effects in the model and BW35 was fitted as a covariate for FI, FC, FE and WG.

We adopted π = 0.9988 in the BayesB model to fit approximately 445 SNP per iteration of the Markov chain comprising 41,000 MCMC samples with the first 1000 samples being discarded. A map file was used to allocate the markers to each of 943 1-Mb non-overlapping windows. Based on previous studies that adopted genomic prediction methodology to perform GWAS [14, 15, 80–82], we investigated the proportion of genetic variance explained by each and every 1-Mb SNP window across the genome. Due to high linkage disequilibrium between the SNPs fitted simultaneously, the QTL effect can be distributed across nearby markers [78]. These previous studies showed that the 1-Mb windows can capture the effects [14, 80–82, 84].

We expect that each window would explain 0.1060% of the genetic variance (100% / 943) in an infinitesimal model as mentioned by Van Goor et al. [80], and windows that explained five times more than expected were considered to be associated with the phenotype. Additionally, we presented the posterior probability of association (PPA) [16] for each associated genomic window, which is the proportion of MCMC samples where the effects of this window were included in the model and accounted for some of the genetic variance [78].

### Overlap with previously mapped QTL

All genomic windows detected were compared with published QTL previously mapped to the chicken genome, using the information available at the Chicken QTLdb - release 35 [7]. The search tool in the Chicken QTLdb website was utilized with QTL coordinates based on the Gallus_gallus-5.0 chicken genome assembly. Additionally, to identify possible pleotropic QTL, we compared the genomic windows detected with QTL previously mapped for fatness traits using the same SNP dataset and the same population (Embrapa F_2_ Chicken Resource Population) [14]. Previously mapped QTL were reported by their respective QTL ID numbers. Genomic windows that did not overlap with previously published QTL regions were considered novel discoveries.

### Identification of positional candidate genes and search for potentially causative SNPs

The genes located within every genomic window that had been shown to be associated with a trait, and their corresponding Gene Ontology terms, were retrieved from the Ensembl Genes 92 database available at Ensembl BioMart [85]. A literature search was conducted to increase or decrease support for the selection of a candidate gene. Using the whole gene list, enrichment analyses were performed by two different approaches: MeSH enrichment to identify enriched MeSH terms using the R/Bioconductor package meshr [18, 86] and, Functional Annotation Tool (FAT) in Database for Annotation, Visualization and Integrated Discovery software (DAVID bioinformatics resources v.6.8, [17, 87]) to identify enriched clusters of genes. To identify MeSh terms and genes enriched clusters, we considered the raw *p*-value < 0.05 and p-value adjusted for multiple testing using the Benjamini & Hochberg [19] procedure (padjusted) < 0.1.

Genes identified in this study were evaluated to determine if they resided within selection signature regions that were previously identified in 28 grandparental chickens (14 TT and 14 CC) that were ancestors of our experimental population [17]. The description of the methods applied to identify the signature selection regions, and the SNP dataset used are available in Boschiero et al. [17].

Additionally, we integrated a sequence SNP dataset from re-sequencing these grandparental animals to identify candidate mutations located in our positional candidate genes. In this study, we only investigated SNPs located in coding regions. To predict whether SNPs that cause changes in amino acids may affect the function of the gene product, we utilized the SIFT (sorting intolerant from tolerant) score to assess the level of conservation in homologous protein sequences using the SIFT algorithm [87] implemented within the VEP tool [86]. High impact SNPs that may cause protein truncation, loss of function or trigger nonsense-mediated decay were also evaluated in the positional candidate genes.

## Supplementary information

**Additional file 1.** The characterization of all the 943 1-Mb SNP windows analyzed including the percentage of the genetic variance explained by each one.

**Additional file 2.** – Genomic windows, associated traits and published overlapping QTL for body weight traits, available at the Chicken QTL database (release 35). The underlined published QTL were previously mapped with microsatellite markers for the same traits in the same population (Embrapa F_2_ Chicken Resource Population).

**Additional file 3.** List with the Ensembl Gene ID and their respective gene names of the 386 genes annotated within the QTL detected.

## Data Availability

The SNPs reported (identified by sequencing) were submitted to dbSNP- NCBI with the handle “LBA_ESALQ”. The datasets used and/or analyzed during the current study (genotypes and phenotypes) are available from the corresponding author on reasonable request.

## Abbreviations

BW1: Body weight at hatch
BW35: Body weight at 35 days of age
BW41: Body weight at 41 days of age
FC: Feed conversion ratio
FE: Feed efficiency
FI: Feed intake
GWAS: Genome-Wide Association Study
MAF: Minor Allele Frequency
MCMC: Markov chain Monte Carlo
PPA: Posterior Probability of Association
QTL: Quantitative Trait Locus
SNP: Single Nucleotide Polymorphism
WG: Weight gain
