# Supplementary material for: Unraveling genomic associations with feed efficiency and body weight traits in chickens through an integrative approach
Source: BMC Genet. 2019 Nov 6;20:83. doi: 10.1186/s12863-019-0783-3 (PMC6836328; doi:10.1186/s12863-019-0783-3)
Supplement: Supplementary file 2 — Additional file 2. – Genomic windows, associated traits and published overlapping QTL for body weight traits, available at the Chicken QTL database (release 35). The underlined published QTL were previously mapped with microsatellite markers for the same traits in the same population (Embrapa F2 Chicken Resource Population). [file 12863_2019_783_MOESM2_ESM.docx]

Additional file 2 – Genomic windows, associated traits and published overlapping QTL for body weight traits, available at the Chicken QTL database (release 35). The underlined published QTL were previously mapped with microsatellite markers for the same traits in the same population (Embrapa F_2_ Chicken Resource Population).

| **GGA_Mb** | **Associated traits** | **Published overlapping QTL^1^** |
| --- | --- | --- |
| 1_54 | BW35, BW41 | QTL:1797, QTL:1807, QTL:1808, QTL:6807, QTL:9406, QTL:13616, QTL:14462, QTL:14467, QTL:17076, QTL:24839, QTL:24848, QTL:24872, QTL:55902, QTL:55908, QTL:55913 |
| 1_55 | BW35 | QTL:1797, QTL:1799, QTL:1807, QTL:1808, QTL:6678, QTL:6807, QTL:6808, QTL:6809, QTL:9406, QTL:9743, QTL:9744, QTL:14467, QTL:17076, QTL:24839, QTL:24848, QTL:24861, QTL:24872, QTL:55902, QTL:55908, QTL:55913 |
| 1_56 | BW35, BW41 | QTL:1797, QTL:1799, QTL:1807, QTL:1808, QTL:6678, QTL:6808, QTL:6809, QTL:9406, QTL:9743, QTL:9744, QTL:17076, QTL:24861, QTL:55902, QTL:55908, QTL:55913 |
| 1_129 | BW35 | QTL:1797, QTL:14355, QTL:14356, QTL:14357, QTL:14358, QTL:17076, QTL:24840, QTL:24849, QTL:24862, QTL:55919, QTL:95410 |
| 1_168 | BW35 | QTL:1797, QTL:1855, QTL:1858, QTL:6579, QTL:6582, QTL:6583, QTL:6584, QTL:7000, QTL:7001, QTL:7002, QTL:7003, QTL:7004, QTL:9750, QTL:9751, QTL:14357, QTL:24873, QTL:66038 |
| 1_181 | BW1 | QTL:1797, QTL:1855, QTL:1858, QTL:6579, QTL:6582, QTL:6583, QTL:6584, QTL:9458, QTL:9497, QTL:9750, QTL:9751, QTL:24873 |
| 2_78 | BW35, BW41 | QTL:6594, QTL:6595, QTL:9752, QTL:55903, QTL:55909 |
| 3_28 | BW35 | QTL:1951, QTL:1957, QTL:1979, QTL:1980, QTL:6599, QTL:6600, QTL:6601, QTL:7167, QTL:7171, QTL:7174, QTL:7180, QTL:17273, QTL:17274, QTL:17275, QTL:17276, QTL:55904, QTL:65713 |
| 3_30 | BW35, BW41 | QTL:1951, QTL:1957, QTL:1979, QTL:1980, QTL:6599, QTL:6600, QTL:6601, QTL:7167, QTL:7171, QTL:7174, QTL:7180, QTL:17273, QTL:17274, QTL:17275, QTL:17276, QTL:55904 |
| 4_69 | BW35 | QTL:1996, QTL:2008, QTL:2015, QTL:2016, QTL:7162, QTL:7181, QTL:7185, QTL:9289, QTL:9300, QTL:9426, QTL:9759, QTL:9760, QTL:11766, QTL:11769, QTL:11773, QTL:14457, QTL:14464, QTL:14470, QTL:17069, QTL:24842, QTL:24855,  QTL:24866, QTL:24875, QTL:24883, QTL:24890, QTL:55905, QTL:55915 |
| 4_74 | BW35, BW41 | QTL:2008, QTL:2015, QTL:2016, QTL:2026, QTL:7157, QTL:7162, QTL:7181, QTL:7185, QTL:9300, QTL:9426, QTL:9759, QTL:9760, QTL:11766, QTL:11769, QTL:11773, QTL:14457, QTL:14464, QTL:14470, QTL:17069, QTL:24842, QTL:24855, QTL:24866, QTL:24875, QTL:24883, QTL:24890 |
| 4_76 | BW35, BW41 | QTL:2008, QTL:2015, QTL:2016, QTL:2026, QTL:7157, QTL:7162, QTL:7181, QTL:7185, QTL:9759, QTL:9760, QTL:11766, QTL:11769, QTL:11773, QTL:14457, QTL:14464, QTL:14470, QTL:17069, QTL:24842, QTL:24855, QTL:24866,  QTL:24875, QTL:24883, QTL:24890, QTL:24949, QTL:30860, QTL:55905, QTL:55915, QTL:62149, QTL:62151, QTL:62157, QTL:62159, QTL:62160, QTL:62162, QTL:62164, QTL:62166, QTL:64510, QTL:64511, QTL:65696,  QTL:65700, QTL:65703, QTL:65706 |
| 6_2 | BW1 | QTL:95421, QTL:95417, QTL:55916 |
| 7_34 | BW35 | QTL:2146, QTL:2158, QTL:2160, QTL:6625, QTL:6935, QTL:7163 |
| 7_36 | BW35 | QTL:2146, QTL:2158, QTL:2160, QTL:6625, QTL:7163 |
| 10_16 | BW41 | QTL:2234, QTL:6945, QTL:7158, QTL:7164, QTL:55907, QTL:55911, QTL:55917 |
| 14_9 | BW35 | QTL:17082, QTL:55925 |
| 24_1 | BW35 | -- |
| 27_3 | BW35, BW41 | QTL:2404, QTL:2405, QTL:2406, QTL:2407, QTL:2408, QTL:2409, QTL:2410, QTL:3356, QTL:6652, QTL:6653, QTL:7159, QTL:7178, QTL:7186, QTL:9521, QTL:9522, QTL:9775, QTL:14459, QTL:14466, QTL:14473, QTL:17084, QTL:24878, QTL:24888, QTL:24892, QTL:55906, QTL:55912, QTL:55918, QTL:55926 |
| 28_0 | BW35, BW41 | QTL:6996, QTL:6958 |

^1^ QTLs ID numbers available at the Chicken QTL database (release 35).
